# 6-Meth­oxy-4-methyl-2*H*-chromen-2-one

**DOI:** 10.1107/S1600536810051652

**Published:** 2010-12-15

**Authors:** Hoong-Kun Fun, Jia Hao Goh, Dongdong Wu, Yan Zhang

**Affiliations:** a X-ray Crystallography Unit, School of Physics, Universiti Sains Malaysia, 11800 USM, Penang, Malaysia; b School of Chemistry and Chemical Engineering, Nanjing University, Nanjing 210093, People’s Republic of China

## Abstract

The whole mol­ecule of the title coumarin derivative, C_11_H_10_O_3_, is approximately planar, with a maximum deviation of 0.116 (3) Å from the least-squares plane defined by all non-H atoms. In the crystal, adjacent mol­ecules are linked into chains along [011] *via* inter­molecular C—H⋯O hydrogen bonds.

## Related literature

For general background to and applications of the title coumarin derivative, see: Grimm & Girard (2006 ▶); Maresca *et al.* (2010 ▶); Parvez & Hadda (2010 ▶); Raj & Wenge (1998 ▶); Yao & Deng (2000 ▶). For related coumarin structures, see: Asad *et al.* (2010 ▶); Saidi *et al.* (2007 ▶). For bond-length data, see: Allen *et al.* (1987 ▶).
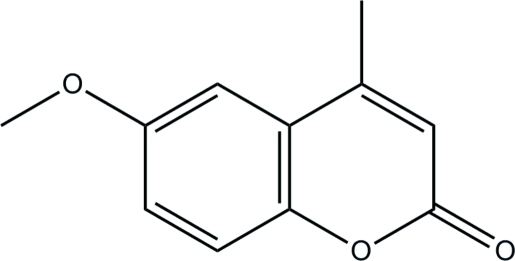


## Experimental

### 

#### Crystal data

C_11_H_10_O_3_
*M* *_r_* = 190.19
Triclinic, 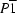

*a* = 7.2554 (2) Å
*b* = 8.0880 (2) Å
*c* = 8.5450 (2) Å
α = 112.988 (1)°
β = 90.234 (1)°
γ = 93.873 (1)°
*V* = 460.31 (2) Å^3^
*Z* = 2
Mo *K*α radiation
μ = 0.10 mm^−1^
*T* = 293 K
0.40 × 0.35 × 0.06 mm

#### Data collection

Bruker SMART APEXII CCD area-detector diffractometer
Absorption correction: multi-scan (*SADABS*; Bruker, 2009 ▶) *T*_min_ = 0.962, *T*_max_ = 0.994
10271 measured reflections
2793 independent reflections
1675 reflections with *I* > 2σ(*I*)
*R*_int_ = 0.021

#### Refinement

*R*[*F* ^2^ > 2σ(*F* ^2^)] = 0.066
*wR*(*F* ^2^) = 0.218
*S* = 1.09
2793 reflections
129 parameters
H-atom parameters constrained
Δρ_max_ = 0.70 e Å^−3^
Δρ_min_ = −0.20 e Å^−3^

### 

Data collection: *APEX2* (Bruker, 2009 ▶); cell refinement: *SAINT* (Bruker, 2009 ▶); data reduction: *SAINT*; program(s) used to solve structure: *SHELXTL* (Sheldrick, 2008 ▶); program(s) used to refine structure: *SHELXTL*; molecular graphics: *SHELXTL*; software used to prepare material for publication: *SHELXTL* and *PLATON* (Spek, 2009 ▶).

## Supplementary Material

Crystal structure: contains datablocks global, I. DOI: 10.1107/S1600536810051652/is2642sup1.cif

Structure factors: contains datablocks I. DOI: 10.1107/S1600536810051652/is2642Isup2.hkl

Additional supplementary materials:  crystallographic information; 3D view; checkCIF report

## Figures and Tables

**Table 1:** Hydrogen-bond geometry (Å, °)

*D*—H⋯*A*	*D*—H	H⋯*A*	*D*⋯*A*	*D*—H⋯*A*
C8—H8*A*⋯O2^i^	0.93	2.56	3.471 (2)	165

Symmetry code: (i) 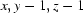.

**Fig. 1.**
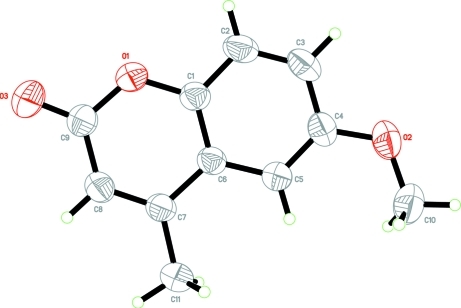
The molecular structure of the title coumarin derivative, showing the atomic numbering scheme. Displacement ellipsoids for non-hydrogen atoms are drawn at the 50 % probability level.

**Fig. 2.**
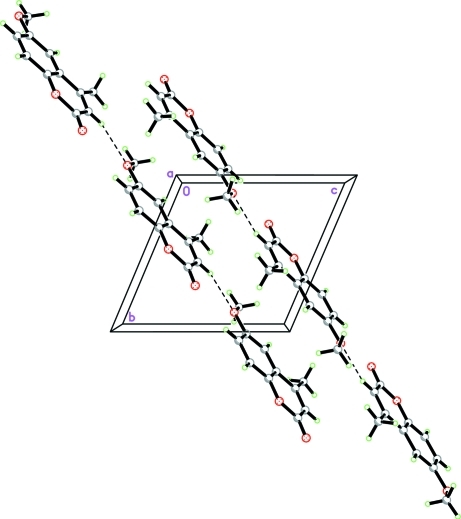
The crystal structure of the title compound, viewed along the a axis, showing one-dimensional chains propagating along the [011] direction. Intermolecular hydrogen bonds are shown as dashed lines.

## supplementary crystallographic information

### Comment

Coumarin is the mother-nuclear structure of many natural products and the importance of coumarin and its analogous compounds which exhibit useful pharmaceutical activities are well-known. Some substituted coumarin and their derivatives have been reported as food constituents, anti-oxidants, stabilizers, immunomodulatory substances, inhibitors of some enzymes, fluorescent markers in analysis, lasers, and in clinical use (Parvez & Hadda, 2010; Maresca *et al.*, 2010; Grimm & Girard, 2006). In addition, 4-substituted coumarins have shown many pharmaceutical activities such as anti-bacterial, anti-fungal, anthelmintic, insecticidal, hypnotic and other biological activities, and most precisely 4-methyl-coumarins have been correlated to several beneficial pharmacological effects too (Yao & Deng, 2000; Raj & Wenge, 1998). In view of the importance of the coumarin derivatives, the crystal structure of the title compound is reported in this paper.

The title coumarin derivative (Fig. 1) has an approximately planar molecular structure, with the methoxy-O atom (C10) deviating -0.116 (3) Å from the least-squares plane defined by all non-hydrogen atoms. All bond lengths (Allen *et al.*, 1987) and angles are within normal ranges and are comparable to those related coumarin structures (Asad *et al.*, 2010; Saidi *et al.*, 2007). In the crystal packing (Fig. 2), adjacent molecules are linked into one-dimensional chains propagating along the [011] direction *via* intermolecular C8—H8A···O2 hydrogen bonds.

### Experimental

The title compound was obtained in the photoreaction of 4-(chloromethyl)-6-methoxy-2*H*-chromen-2-one in visible light. The compound was purified by flash column chromatography. Good quality single crystals suitable for X-ray analysis were obtained from slow evaporation of a 1:1 solution of acetone and petroleum ether.

### Refinement

All hydrogen atoms were placed in their calculated positions, with C—H = 0.93 or 0.96 Å, and refined using a riding model, with *U*_iso_(H) = 1.2 or 1.5*U*_eq_(C). The rotating group model was applied to the methyl groups.

### Crystal data

**Table:**

C_11_H_10_O_3_	*Z* = 2
*M**_r_* = 190.19	*F*(000) = 200
Triclinic, *P*1	*D*_x_ = 1.372 Mg m^−^^3^
Hall symbol: -P 1	Mo *K*α radiation, λ = 0.71073 Å
*a* = 7.2554 (2) Å	Cell parameters from 3241 reflections
*b* = 8.0880 (2) Å	θ = 2.6–30.0°
*c* = 8.5450 (2) Å	µ = 0.10 mm^−^^1^
α = 112.988 (1)°	*T* = 293 K
β = 90.234 (1)°	Plate, yellow
γ = 93.873 (1)°	0.40 × 0.35 × 0.06 mm
*V* = 460.31 (2) Å^3^	

### Data collection

**Table:**

Bruker SMART APEXII CCD area-detector diffractometer	2793 independent reflections
Radiation source: fine-focus sealed tube	1675 reflections with *I* > 2σ(*I*)
graphite	*R*_int_ = 0.021
φ and ω scans	θ_max_ = 30.6°, θ_min_ = 2.6°
Absorption correction: multi-scan (*SADABS*; Bruker, 2009)	*h* = −10→10
*T*_min_ = 0.962, *T*_max_ = 0.994	*k* = −11→11
10271 measured reflections	*l* = −11→12

### Refinement

**Table:**

Refinement on *F*^2^	Primary atom site location: structure-invariant direct methods
Least-squares matrix: full	Secondary atom site location: difference Fourier map
*R*[*F*^2^ > 2σ(*F*^2^)] = 0.066	Hydrogen site location: inferred from neighbouring sites
*wR*(*F*^2^) = 0.218	H-atom parameters constrained
*S* = 1.09	*w* = 1/[σ^2^(*F*_o_^2^) + (0.0991*P*)^2^ + 0.086*P*] where *P* = (*F*_o_^2^ + 2*F*_c_^2^)/3
2793 reflections	(Δ/σ)_max_ < 0.001
129 parameters	Δρ_max_ = 0.70 e Å^−^^3^
0 restraints	Δρ_min_ = −0.20 e Å^−^^3^

### Special details

**Table:**

Geometry. All esds (except the esd in the dihedral angle between two l.s. planes) are estimated using the full covariance matrix. The cell esds are taken into account individually in the estimation of esds in distances, angles and torsion angles; correlations between esds in cell parameters are only used when they are defined by crystal symmetry. An approximate (isotropic) treatment of cell esds is used for estimating esds involving l.s. planes.
Refinement. Refinement of F^2^ against ALL reflections. The weighted R-factor wR and goodness of fit S are based on F^2^, conventional R-factors R are based on F, with F set to zero for negative F^2^. The threshold expression of F^2^ > 2sigma(F^2^) is used only for calculating R-factors(gt) etc. and is not relevant to the choice of reflections for refinement. R-factors based on F^2^ are statistically about twice as large as those based on F, and R- factors based on ALL data will be even larger.

### Fractional atomic coordinates and isotropic or equivalent isotropic displacement parameters (Å^2^)

**Table:**

	*x*	*y*	*z*	*U*_iso_*/*U*_eq_	
O1	0.19469 (16)	0.53037 (17)	0.87871 (16)	0.0508 (4)	
O2	0.6197 (2)	1.09931 (18)	1.34611 (17)	0.0638 (4)	
O3	0.1468 (2)	0.2933 (2)	0.63843 (19)	0.0679 (5)	
C1	0.3087 (2)	0.6718 (2)	0.9894 (2)	0.0417 (4)	
C2	0.2327 (3)	0.7819 (3)	1.1396 (2)	0.0512 (5)	
H2A	0.1101	0.7603	1.1617	0.061*	
C3	0.3402 (3)	0.9229 (3)	1.2551 (2)	0.0527 (5)	
H3A	0.2901	0.9974	1.3559	0.063*	
C4	0.5250 (3)	0.9555 (2)	1.2222 (2)	0.0472 (4)	
C5	0.5999 (2)	0.8469 (2)	1.0726 (2)	0.0438 (4)	
H5A	0.7225	0.8696	1.0510	0.053*	
C6	0.4917 (2)	0.7016 (2)	0.9521 (2)	0.0384 (4)	
C7	0.5603 (2)	0.5807 (2)	0.7919 (2)	0.0414 (4)	
C8	0.4450 (2)	0.4441 (2)	0.6875 (2)	0.0463 (4)	
H8A	0.4895	0.3663	0.5851	0.056*	
C9	0.2564 (3)	0.4127 (2)	0.7264 (2)	0.0475 (4)	
C10	0.8119 (3)	1.1270 (3)	1.3272 (3)	0.0695 (6)	
H10C	0.8624	1.2287	1.4242	0.104*	
H10D	0.8317	1.1495	1.2260	0.104*	
H10A	0.8718	1.0217	1.3187	0.104*	
C11	0.7558 (3)	0.6091 (3)	0.7459 (3)	0.0573 (5)	
H11D	0.7824	0.5119	0.6419	0.086*	
H11A	0.8387	0.6128	0.8352	0.086*	
H11B	0.7713	0.7209	0.7311	0.086*	

### Atomic displacement parameters (Å^2^)

**Table:**

	*U*^11^	*U*^22^	*U*^33^	*U*^12^	*U*^13^	*U*^23^
O1	0.0352 (6)	0.0577 (8)	0.0514 (8)	0.0001 (5)	0.0021 (5)	0.0132 (6)
O2	0.0694 (10)	0.0531 (8)	0.0488 (8)	0.0036 (7)	−0.0011 (7)	−0.0017 (6)
O3	0.0523 (8)	0.0717 (9)	0.0622 (9)	−0.0130 (7)	−0.0092 (7)	0.0103 (8)
C1	0.0368 (8)	0.0467 (9)	0.0413 (9)	0.0056 (7)	0.0016 (7)	0.0164 (7)
C2	0.0411 (9)	0.0600 (11)	0.0516 (11)	0.0141 (8)	0.0130 (8)	0.0193 (9)
C3	0.0579 (11)	0.0553 (11)	0.0413 (10)	0.0187 (9)	0.0120 (8)	0.0125 (8)
C4	0.0541 (11)	0.0434 (9)	0.0394 (9)	0.0087 (8)	−0.0003 (8)	0.0104 (7)
C5	0.0400 (9)	0.0467 (9)	0.0416 (9)	0.0039 (7)	0.0031 (7)	0.0139 (7)
C6	0.0362 (8)	0.0418 (8)	0.0367 (8)	0.0073 (6)	0.0023 (6)	0.0141 (7)
C7	0.0384 (9)	0.0457 (9)	0.0387 (9)	0.0068 (7)	0.0042 (7)	0.0145 (7)
C8	0.0461 (10)	0.0480 (9)	0.0388 (9)	0.0059 (7)	0.0031 (7)	0.0102 (7)
C9	0.0429 (9)	0.0511 (10)	0.0440 (10)	0.0006 (8)	−0.0033 (7)	0.0144 (8)
C10	0.0654 (14)	0.0596 (12)	0.0646 (14)	−0.0073 (10)	−0.0111 (11)	0.0060 (10)
C11	0.0466 (10)	0.0633 (12)	0.0508 (11)	0.0045 (9)	0.0119 (8)	0.0100 (9)

### Geometric parameters (Å, °)

**Table:**

O1—C9	1.377 (2)	C5—H5A	0.9300
O1—C1	1.381 (2)	C6—C7	1.450 (2)
O2—C4	1.367 (2)	C7—C8	1.348 (2)
O2—C10	1.417 (3)	C7—C11	1.500 (2)
O3—C9	1.206 (2)	C8—C9	1.438 (3)
C1—C2	1.387 (2)	C8—H8A	0.9300
C1—C6	1.394 (2)	C10—H10C	0.9600
C2—C3	1.370 (3)	C10—H10D	0.9600
C2—H2A	0.9300	C10—H10A	0.9600
C3—C4	1.400 (3)	C11—H11D	0.9600
C3—H3A	0.9300	C11—H11A	0.9600
C4—C5	1.376 (2)	C11—H11B	0.9600
C5—C6	1.407 (2)		
			
C9—O1—C1	121.54 (14)	C8—C7—C11	121.52 (15)
C4—O2—C10	117.77 (15)	C6—C7—C11	119.97 (15)
O1—C1—C2	116.85 (15)	C7—C8—C9	123.28 (16)
O1—C1—C6	121.51 (15)	C7—C8—H8A	118.4
C2—C1—C6	121.65 (16)	C9—C8—H8A	118.4
C3—C2—C1	119.36 (17)	O3—C9—O1	116.69 (17)
C3—C2—H2A	120.3	O3—C9—C8	126.37 (18)
C1—C2—H2A	120.3	O1—C9—C8	116.95 (15)
C2—C3—C4	120.41 (16)	O2—C10—H10C	109.5
C2—C3—H3A	119.8	O2—C10—H10D	109.5
C4—C3—H3A	119.8	H10C—C10—H10D	109.5
O2—C4—C5	124.24 (17)	O2—C10—H10A	109.5
O2—C4—C3	115.58 (16)	H10C—C10—H10A	109.5
C5—C4—C3	120.18 (17)	H10D—C10—H10A	109.5
C4—C5—C6	120.30 (16)	C7—C11—H11D	109.5
C4—C5—H5A	119.8	C7—C11—H11A	109.5
C6—C5—H5A	119.8	H11D—C11—H11A	109.5
C1—C6—C5	118.10 (15)	C7—C11—H11B	109.5
C1—C6—C7	118.22 (15)	H11D—C11—H11B	109.5
C5—C6—C7	123.68 (15)	H11A—C11—H11B	109.5
C8—C7—C6	118.51 (15)		
			
C9—O1—C1—C2	−179.81 (14)	C2—C1—C6—C7	−179.76 (15)
C9—O1—C1—C6	−0.2 (3)	C4—C5—C6—C1	−0.1 (3)
O1—C1—C2—C3	179.14 (15)	C4—C5—C6—C7	−179.79 (14)
C6—C1—C2—C3	−0.4 (3)	C1—C6—C7—C8	−0.8 (2)
C1—C2—C3—C4	−0.1 (3)	C5—C6—C7—C8	178.89 (15)
C10—O2—C4—C5	−6.8 (3)	C1—C6—C7—C11	179.31 (16)
C10—O2—C4—C3	173.65 (17)	C5—C6—C7—C11	−1.0 (3)
C2—C3—C4—O2	−179.84 (16)	C6—C7—C8—C9	0.4 (3)
C2—C3—C4—C5	0.6 (3)	C11—C7—C8—C9	−179.67 (17)
O2—C4—C5—C6	−179.97 (15)	C1—O1—C9—O3	179.91 (15)
C3—C4—C5—C6	−0.4 (3)	C1—O1—C9—C8	−0.2 (3)
O1—C1—C6—C5	−178.98 (14)	C7—C8—C9—O3	179.99 (18)
C2—C1—C6—C5	0.6 (3)	C7—C8—C9—O1	0.1 (3)
O1—C1—C6—C7	0.7 (2)		

### Hydrogen-bond geometry (Å, °)

**Table:**

*D*—H···*A*	*D*—H	H···*A*	*D*···*A*	*D*—H···*A*
C8—H8A···O2^i^	0.93	2.56	3.471 (2)	165

Symmetry codes: (i) *x*, *y*−1, *z*−1.
